# A Review of *Rhazya stricta* Decne Phytochemistry, Bioactivities, Pharmacological Activities, Toxicity, and Folkloric Medicinal Uses

**DOI:** 10.3390/plants10112508

**Published:** 2021-11-19

**Authors:** Abdulaziz Albeshri, Nabih A. Baeshen, Thamer A. Bouback, Abdullah A. Aljaddawi

**Affiliations:** 1 Department of Biological Science, Faculty of Science, King Abdul-Aziz University, Jeddah 21589, Saudi Arabia; nabih_baeshen@hotmail.com (N.A.B.); tbouback@kau.edu.sa (T.A.B.); aaljaddawi@kau.edu.sa (A.A.A.); 2 Princess Dr. Najla Bint Saud Al-Saud Center for Excellence Research in Biotechnology, King Abdul-Aziz University, Jeddah 21589, Saudi Arabia

**Keywords:** *Rhazya stricta*, folkloric medicine, alkaloid, phytochemicals, pharmacological activity, toxicity

## Abstract

The local medicinal plant *Rhazya stricta Decne* is reviewed for its folkloric medicinal, phytochemical, pharmacological, biological, and toxicological features. *R. stricta* has been used widely in different cultures for various medical disorders. The phytochemical studies performed on the *R. stricta* extract revealed many alkaloidal and fatty acid compounds. Moreover, several flavonoid and terpenoid compounds were also detected. Pharmacological activates of *R. stricta* extracts are approved to possess antimicrobial, antioxidant, anticancer, antidiabetic, and antihypertensive activities. Additionally, *R. stricta* extract was found to hold biological activates such as larvicidal and phytoremediation activates *R. stricta* extract was found to be toxic, genotoxic, and mutagenic. *R. stricta* contains novel phytochemical compounds that have not been investigated pharmacologically. Further research is needed through in vitro and in vivo experiments to pave the road for these compounds for medical, veterinary, and ecological uses.

## 1. Introduction

*R. stricta* is one of the most economically valuable medicinal plants found throughout arid South Asia and the Arabian Peninsula. Leaf extracts were traditionally utilized in the treatment of a wide variety of illnesses, such as syphilis, parasitic infections, hyperglycemia, and rheumatism, as well as the common cold [1]. The *Rhazya stricta* species was labeled after one Muslim scientist known as Mohammed bin Zakariya AlRazi (925), and it is generally recognized in Europe as Rhazes [2]. Numerous studies used various parts of *R. stricta* extract to screen for phytochemical constituents. Over a hundred alkaloids and many compounds belonging to other groups, such as flavonoids and lipids, have been isolated [3,4]. It has been proven that the alkaloidal compounds possess multiple activities, including antitumor, antimicrobial, and antihypertensive [5]. The main objective of this review is to provide advanced and updated information about *R.stricta* plant research. 

## 2. Methodology

The content for this review was extracted from Google Scholar articles. The scientific name *“Rhazya stricta”* was used to cover all relevant data from 1800–2021, including traditional uses, nutritional compositions, phytochemical compounds, and pharmacological properties (anticancer, antioxidant, antiviral, antimicrobial, anti-inflammatory, hepatoprotective, antidiabetic, and antihypertensive) of the plant described in this review.

## 3. Regional Names

In Urdu, it is referred to as “Rangobul,” “Vergalum,” “Ganderi” in Pushto, and “harmal” in Arabic. It is important, however, to distinguish the harmal for *Peganum harmala* from the harmal for *R. stricta* [2].

## 4. Regional Distribution

**Figure 1:**
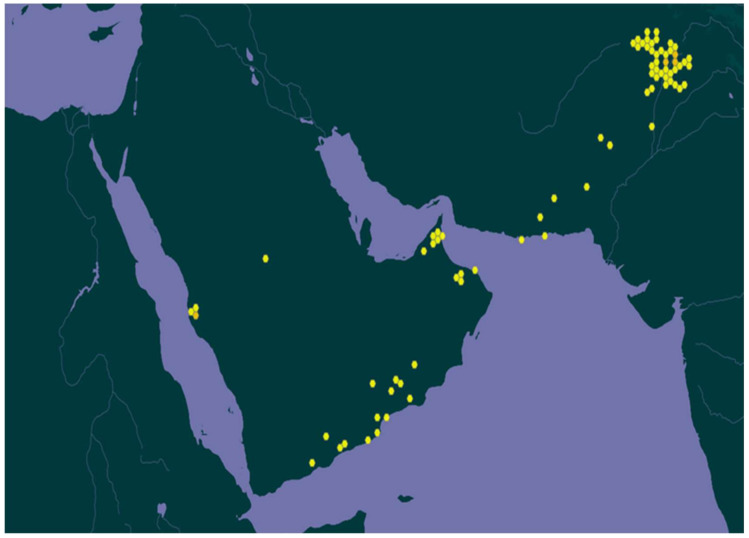
Map shows the regional distribution of the *R. stricta* plant [7].

*R. stricta* is distributed throughout Southwest Asia (India, Pakistan, and Afghanistan) and the countries bordering the Arabic gulf, e.g., Saudi Arabia, the United Arab Emirates (UAE), Iraq, Iran, and Qatar [6] (Figure 1).

## 5. *R. Stricta* Taxonomy

*R. stricta* is a species belonging to the *Apocynaceae* family. The *Apocynaceae* family contains 424 genera and over 4600 species, which are classified into five subfamilies: *Apocynoideae*, *Periplocoideae*, *Rauvolfioideae*, *Asclepiadoideae*, and *Secamonoideae* [8] (Table 1).

## 6. Morphology Characteristics

**Figure 2:**
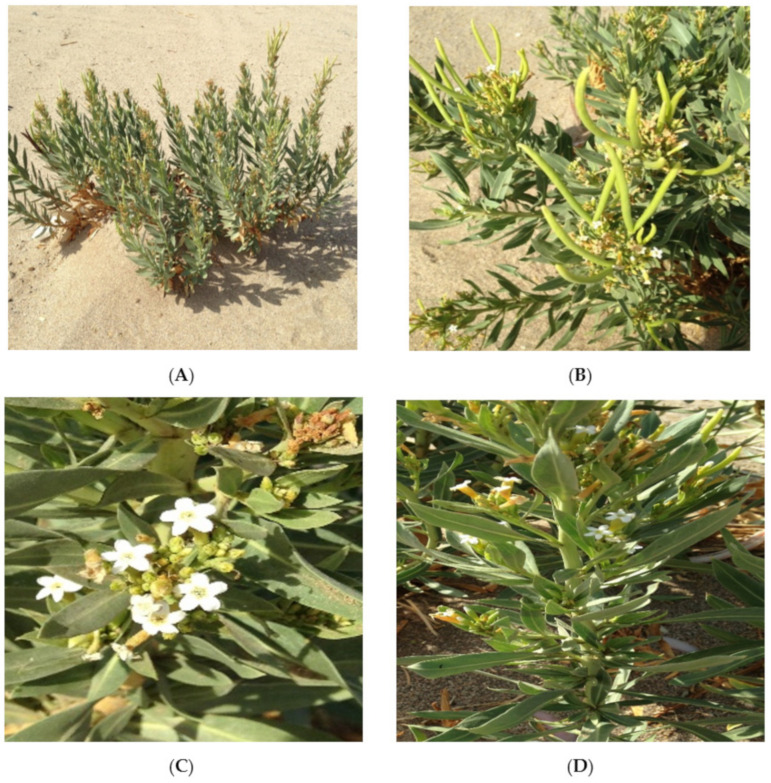
Photos captured of (**A**) whole plant of *R. stricta* or its parts (**B**) fruits, (**C**) flowers, and (**D**) stem and leaves from Wadi Fatimah, Makkah.

*R. stricta* is an evergreen miniature shrub with thick foliage. It is a glabrous, upright perennial plant with many branches emerging from the base. The main stalk is smooth and thickly branched, particularly towards the base, in a semi-erect form. Sessile and simple leaf are linear-oblong or ellipsoidal, practically upright, with an entire border and sharp apex, dense, leather, and parallel blade tapering toward the base. Flowers are bisexual; inflorescences are axillary cymes found near the tips of branches; flowers are pentamerous, white, 2–2.5 cm long, short-pedicelled, and have inserted stamens; Flowers are heterosexual; inflorescences are axillary cymes located towards the ends of branches; flowers are pentamerous, white, 2–2.5 cm in length, short-pedicelled, and have attached stamens; flowers have white petals; the calyx is roughly 4 mm long, deeply lobed. The corolla is 1–1.4 cm in diameter, white; tube cylindrical; lobes ovate, with a rounded mucronate apex, c. 12–15 mm long, and have a brownish-green tube enlarged slightly above the middle and longer than the salverform limb, partially occluded by bristles at the throat; limb lobes are widely obovate, obtuse, mucronate, and are white inside [4] (Figure 2).

## 7. Folkloric Medicinal Uses of *R. Stricta*

*R. stricta* leaves are exploited in the traditional system of medicine in rural areas of Saudi Arabia to treat syphilis, chronic rheumatism, and body pain. [8]. Local folk medicine practitioners utilize *R. stricta* to treat type 2 diabetes, certain inflammatory disorders, helminthiasis, and sore throat [9,10]. In Pakistan, *R. stricta* extract used to treat pimples and acne on the face. Additionally, fresh leaves are preserved in footwear and placed under the soles to alleviate foot burn and treat rheumatic disorders. Healers in Oman treat chest pain, conjunctivitis, constipation, and a variety of other ailments with *R. stricta* [11,12]. 

## 8. Content Properties of *R. stricta* Extract

**Figure 3:**
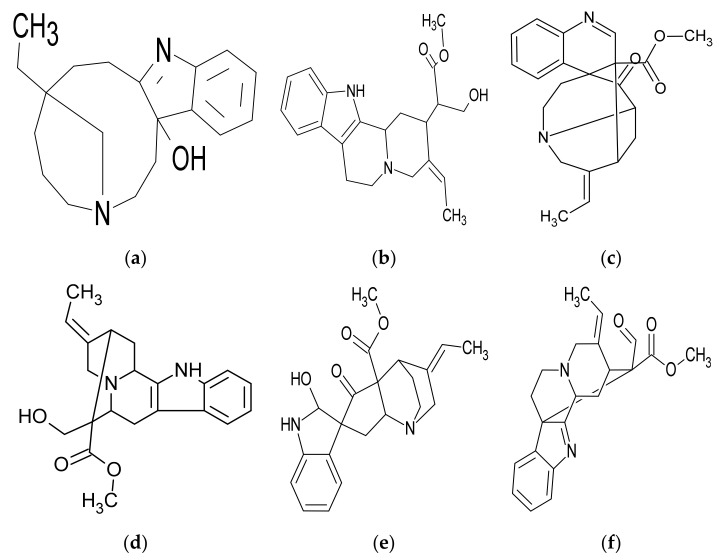
Chemical structures of some alkaloidal compounds (**a**) Rhazidigenine, (**b**) Rhazimanine, (**c**) Rhazimine, (**d**) Rhazine, (**e**) Rhazicine, and (**f**) Rhazimal named after the *R. stricta* plant [29].

Several studies have exerted their efforts on discovering the alkaloid compounds, and they also discovered some non-alkaloid compounds [13,14,15,16,17,18,19,20,21,22,23,24] (Figure 3). A study estimated the contents of *R. stricta* extracts; the root contains the most alkaloids (3.5 g/100 g), while the leaves contain the most tannins, phenolic compounds, and antioxidants, (0.64 g/100 g), (1.4 g/100 g), and (0.56 g/100 g), respectively. The stems contain a high concentration of flavonoids (0.74 g/100 g) [25]. Another study examined the metal content of *R. stricta* extract and discovered the presence of several metals, including Fe, Cd, Ar, Ma, Ca, Cr, Cd, Ni, Pb, K, Na, and Cu [26]. A study determined that *R. stricta* is rich in a variety of alkaloids, flavonoids, polyphenols, tannins, and many other phytochemicals by analyzing the functional groups identified in the root extract [27]. Saponins, tannins, alkaloids, flavonoids, and polyphenols were detected in aqueous extract *R. stricta* during the phytochemical analysis [28] (Table 2 and Table 3).

## 9. Toxicity Studies of *Rhazya stricta*

### 9.1. Toxicity In Vivo Experiments

*Rhazya stricta* leaf was evaluated for its toxicity to Najdi lambs. After oral administration (1 g/kg/d), body weight loss, ruminal, diarrhea, breathlessness, and hind limb weakness were observed. Kidney disease, pulmonary edema, internal bleeding and lung damage, lymphocytes in essential organs, and cardiac vessel congestion were linked to increases in serum AST and LDH, increased bilirubin and urea elevated levels, decreased protein content, albumin, and calcium levels, and leucopenia and anemia [38]. Adult albino rats received intraperitoneal administration (15 mg/kg body weight) of *R. stricta* extract that significantly decreased their total number of white blood cells. The following day after injection, there was a substantial drop, and within three days, there was a 50–60% reduction. The blood cell count recovered to normal after 7–10 days. In dogs, an intravenous dose of the extract (80 mg/kg) resulted in acute salivation and rigor, followed by respiratory depression, convulsions, and fatality within 15 min [39]. The LD50 of *R. stricta* extract was determined to be (16.0 g/kg) when given orally to mice. At the relatively increased doses used, the plant extract did not cause death and did not appear to be toxic [10]. Another research evaluated the influence of *R. stricta* extract on the growth of rat fetuses. The extract concentration (0.5–2 g/kg/day) for 3 days in pregnancy had no significant influence on abnormalities. Except for a generalized reduction of growth, no skeletal abnormalities were detected. Increased dosages (5 or 8 g/kg/day for 3 days) decreased the percentage of viable fetuses and affected placental development, potentially contributing to the reported intrauterine development abnormalities and fetal mortality [40].

### 9.2. Genotoxicity and Mutagenicity

Through the comet assay, significant increases in genotoxicity were observed for *R. stricta* extract at 10 mg/mL doses at different time points. Mutagenicity was tested by using Ames Salmonella assay. *R. stricta* was determined not to be mutagenic to *Salmonella typhimurium* (TA100) and *Salmonella typhimurium* (TA98) [41]. Saccharomyces cerevisiae suspensions were exposed to increasing amounts of aqueous extract of the *R. stricta* leaf. The extract was found to have significant lethal and mutagenic activity. As the concentration or duration of exposure increased, the survival percentage decreased [42]. Another study tested three *R. stricta* extracts by administering them to rats via oral gavage independently, and the three extracts were whole aqueous, alkaloid, and nonalkaloid. The results suggested that whole aqueous and alkaloid extracts of *R. stricta* altered the genomic randomly amplified polymorphic DNA profile, induced significant DNA damage, increased the formation of micronuclei, induced chromosomal aberrations, and decreased the mitotic index [43].

## 10. Pharmacological Activities of *R. stricta*

### 10.1. Antibacterial Activity

In vitro, R. stricta leaf and fruit extract demonstrated antibacterial activity against Staphlococcus aureus, Escherichia coli, Pseudomonasaeruginosa, Bacillus subtilis, Streptococcus pyogenes, and Salmonella typhi [44]. R. stricta leaf extract demonstrated a control of bacterial growth on locally isolated meningococcal strains that increased with concentration and treatment time [45]. Chloroformic and methanolic extracts of R. stricta roots exhibited antimicrobial activity toward B. subtilis, E. coli, S. aureus, and P. aeruginosa. Tetrahydrosecamine was purified from the plant and demonstrated a wide range of antibacterial activity (effective toward all bacteria except E. coli; MIC values ranged from 0.1 to 5.0 mg/mL). Another active substance, strictanol, was also shown to be effective against P. aeruginosa and E. coli (MIC 0.5 mg/mL for both microbes) [46]. The Ag nanoparticles synthesized using silver nitrate and methanol root extract of R. stricta showed improved antibacterial activity against B. subtilis and E. coli [47]. At low concentrations, the tested R. stricta extract mixed with Ag nanoparticles inhibited the growth of several pathogenic bacteria, including Klebsiella pneumoniae, B. subtilis, and S. typhi [31]. The antibacterial activity of five R. stricta leaf extracts at various concentrations was examined against a board of gram-negative and gram-positive bacteria. R. stricta organic alkaloid extract was most effective against E. coli and methicillin-resistant Staphylococcus aureus (MRSA), resulting in the disruption of cell membranes [48]. Acetone and the methanolic extract of R. stricta leaves demonstrated antibacterial activity against Propionibacterium acnes at a (50 mg/mL) minimum inhibitory concentration and zone of inhibition 25.6 ± 1.94 mm [49]. The biogenic Au nanoparticles and R. stricta extract degraded the membrane of E. coli (MIC1 425.0 mg/mL) and B. subtilis (50.0 mg/mL), and stimulated the production of reactive oxygen species, resulting in the death of microbial cells [50]. R. stricta extract suppressed methicillin-resistant Staphylococcus aureus (MRSA) growth, with zones of inhibition extending from 6 to 19 mm, and transmission electron microscopy demonstrated that the extract alters MRSA bacteria cellular architecture [51].

### 10.2. Antifungal Activity

*R. stricta* chloroformic and methanolic root fractions demonstrated antifungal activities against *Aspergillus terreus*, *Aspergillus flavus*, and *Candida albicans* [46]. Another study revealed that fractionated *R. stricta* methanol and chloroform samples showed antifungal activity against *Trichophyton longifusis*, C. albicans, A. flavus, and *Fusarium solani* [52].

### 10.3. Antioxidant Activity

At some doses, *R. stricta* extract exhibits antioxidant effects in rats by increasing glutathione levels and decreasing lipid peroxidation [53]. In comparison to the tocopherol drug and the synthetic antioxidant butylated hydroxyanisole, *R. stricta* methanolic extract was a significant source of natural antioxidants with high free radical scavenging and anion radical scavenging potentials [54]. Significant lipoxygenase and acetylcholinesterase inhibitory activity were observed by the ethanolic extract of *R. stricta* fruit [44]. To determine the impact of the climate conditions on *R. stricta*, the plant leaves collected from Riyadh and the western region were extracted. Both extracts exhibited antioxidant activity, with significant superior performance to *R. stricta* leaves collected from the western region by six evaluation of superoxide radical scavenging and scavenging of hydrogen peroxide levels [4]. The antioxidant activity of root fractions of *R. stricta* was determined using a variety of antioxidant assays. The fractions obtained by solvent-solvent extraction of *R. stricta* root raw extract exhibited remarkable free radical scavenging activity, with an IC50 of 400–776 g/mL [55].

### 10.4. Anticancer Activity

Tetrahydrosecamine diol, which was identified in *R. stricta,* possesses remarkable anticancer activity in vitro against KB carcinoma of the nasopharynx with an ED_50_ 0.0038 µg/mL [56]. Rhazinilam, which mimics taxol cellular activity by suppressing both microtubule assembly and disassembly in vitro, supported the formation of abnormal tubulin spirals and resulted in the formation of microtubule bundles, multiple asters, and microtubule constancy at low temperatures. In vitro, rhazinilam was cytotoxic to a wide range of cancer cell lines at low micromolar concentrations, but it displayed no activity in vivo [57]. *R. stricta* ethanol extract induced apoptosis in breast cancer cells by inhibiting cellular growth and colony formation, stating that it may be a beneficial chemo-preventive or drug product in the treatment of breast cancer [58]. Treatment of MDA-MB-231 cells with *R. stricta* fruit ethyl acetate fraction increased p53, Bax, and caspase 3/7 expression and activation. A cell migration scratch assay indicated that the extract at non-cytotoxic concentrations inhibited the highly invasive MDA-MB-231 cell lines migration. Additionally, RT-PCR analysis revealed significantly decreased (MMP-2) and (MMP-9) expression, both of which play a critical role in breast cancer metastasis. Breast tissue histological assessments in experimental animals revealed a slight improvement in tissue treated with fruit ethyl acetate fraction [32]. On HepG2 and Caco cells, the ethanol extract of *R. stricta* was highly effective (IC50 values of 25 µg/mL and 35 µg/mL, respectively) [59]. Additionally, an in vivo study established experimental evidence by measuring serum liver enzymes and the histopathological alteration of liver tissue for the methanol extract of *R. stricta* aerial parts antitumor efficacy against hepatocellular carcinoma. This effect may be a result of the compound’s hepatoprotective properties, antiproliferative activity, and antiangiogenic potential [60]. The crude alkaloid extract of *R. stricta* significantly induced apoptosis in pancreatic cancer cells with IC50 (78.77 and 41.4 µg/mL) on PANC-1 and AsPC-1 cell lines [61]. The ethanol extract of *R. stricta* leaves suppressed colony formation development in HepG2 cells and significantly restricted cell cycle in the G2/M phase 12 and 48 h following administration, as well as substantial limitation at the G1/S phase after 24 h. This finding supports the use of *R. stricta* as a novel anticancer agent in the treatment of hepatocellular carcinoma [62]. The compounds Epi-rhazyaminine, 20-epi-sitsirikine, eburenine, strictamine, (16R)-Eisositsirikine, antirhine, and strictanol were identified and tested using the MTT assay targeting three types of cancer cells (HCT-116, PC-3, and HepG2) as well as a single kind of normal cell (VERO). The phytochemicals studied had a weak cytotoxic effect on the three cancer cell lines [36]. During 24 and 48 h period assays, *R. stricta* nanoparticles had a substantial inhibitory impact on Hep G-2 cell viability at concentrations of 100 and 500 g/mL [63].

### 10.5. Antidiabetic Activity

*R. stricta* water extract showed no noticeable impact on a glucose concentration introduced orally to rats with and without diabetes. Frequent treatment of *R. stricta* in a water supply had no effect on the glucose homeostasis measures investigated (plasma glucose, body mass, feed and fluid intake, and blood fructosamine) during a 37-day period in either the normal or diabetic stage of this study [64]. The acute administration of the lyophilized *R. stricta* extract to rats at a dosage of 4 g/kg resulted in an important increase in insulin concentration. *R. stricta* at a dosage of 8 g/kg significantly decreased plasma glucose concentrations at 0.5 and 1 h after treatment in streptozotocin-diabetic rats loaded orally with glucose (1 g/kg). Chronic administration with a lyophilized extract of *R. stricta* to mice and rats for 28 days did not impact plasma, glucose, or insulin concentrations or on any of the hematological or biochemical parameters examined [65]. *R. stricta* extract was administered orally to diabetic rats at dosages of (0.5, 20, and 4.0 g/kg) and the glucose level was significantly lowered 1 h (2 and 4 g/kg) and 2 h (4 g/kg) after the extract was administered. This was followed by substantial increases in insulin concentrations 1, 2, and 4 h after the extract was administered at dosages of (2 and 4 g/kg). Combined administration of hyperglycemic rats with the leaf extract (0.5, 20 and 5.0 g/kg) and glibenclamide (5.0 mg/kg) substantially increased the effects of the extract or glibenclamide on glucose, insulin, and glucagon when used alone. When the leaf extract was administered at dosages of (0.5, 2, and 4 g/kg) daily for six successive days, the glucose level decreased by about 6, 8, and 30%, respectively [66]. The effects of *R. stricta* extract on adiponectin concentrations could be beneficial in the treatment of diabetes by increasing the adiponectin level [67]. *R. stricta* root extract demonstrated significant antidiabetic activity by inhibiting Dipeptidyl peptidase-IV (up to 61%) and β-secretase (up to 83%) enzymes, resulting in an increase in glucagon-like peptide-1 secretion [27]. The ethyl acetate fraction of *R. stricta* is most effective at lowering blood glucose amounts in fasting and random conditions, and the lowering of blood glucose levels was similar to that of Glucophage, a basic antidiabetic drug [68].

### 10.6. Other Pharmacological Activities

The lyophilized extract of *R. stricta* (5–100 mg/kg) concentrations had a variable impact on heart rate and a dose-dependent reduction in mean blood pressure in urethane-anaesthetized rats [65]. The potential that part of the *R. stricta* extract stated therapeutic effects are related to its immunomodulatory capacity was explored in one experiment using ex vivo generation of macrophage-derived cytokines in mice. Every mouse was treated twice weekly with an alkaloidal portion of *R. stricta* (0.5 and 1.0 mg/individual). Peritoneal cells were extracted, grown, and tested for IL-1a and TNF using an enzyme-linked immunosorbent assay (ELISA). *R. stricta*’s alkaloidal portion considerably enhanced the secretion of these two proinflammatory cytokines [69]. When compared to other UAE medicinal plants, *R. stricta* demonstrated the strongest ability to relax smooth muscles, implying that the herb may have antispasmodic capabilities. This appears to corroborate the plant’s folk medicinal use in certain regions [3]. The chloroform stem extract of *R. stricta* can stimulate early neuronal differentiation in stem cells and may possess a potential therapeutic agent for neurodegenerative diseases [70]. The methanol extract of *R. stricta* significantly reduced the degree and frequency of diarrhea in rats caused by castor oil. Moreover, *R. stricta* extract significantly reduced castor oil-induced intestinal transit by 24.44% at a dosage of (250 mg/kg) and 58.88% at a dosage of (500 mg/kg) [71]. The immunomodulatory impact of *R. stricta* methanol extract was investigated by giving it to broiler chicks in their drinking water for two weeks before they were challenged with sheep erythrocytes. Significant increases in phagocytic activity, lymphocyte proliferation, and percentages of circulating lymphocytes were detected, indicating an improvement in cellular immunity. Significant increases in the serum levels of total antibodies of the IgM and IgG isotypes were also seen, indicating an improved humoral response [37]. 

## 11. Biological Activities

The raw extract of *R. stricta* was found to be larvicidal and inhibited growth (8-36%) with increasing doses (200-1000ppm) in *Aedes aegypti* fourth instar larvae [72]. By impairing membrane function and photosynthetic ability, the leaf extract of *R. stricta* inhibits the growth and metabolic activity of *Salsola villosa* [73]. The high growth rate of *R. stricta*, its resistance to heavy toxic metals, and its capacity to absorb and concentrate metals inside the plant all support its application in phytoremediation [74]. With an increase in the concentrations of the leaf extract of the medicinal plant *R. stricta*, the mortality and repellency of *Rhyzopertha dominica* and *Trogoderma granarium* increased. Thus, *R. stricta* may be a useful ingredient in an effective pest control system designed to combat stored grain pests [75]. Dry powdered leaves or succulent shoots of *R. stricta* (30 g/kg of soil), thoroughly mixed with soil 20 days before transplanting, may act as an effective control method against bacterial wilt [76]. *R. stricta* methanolic extract inhibited seed germination of *Phalaris minor*, *Chenopodium album*, and *Rumex dentatus* by percentages of 43%, 47%, and 42%, respectively, in soil. *R. stricta* demonstrated promising allelopathic activity [77]. Water extract of *R. stricta* was applied at concentrations ranging from 100 to 500 parts per million, inhibiting the growth by reducing hatchability of eggs and causing the death of *Culex pipiens* mosquitoes [78]. *R. stricta* extract also demonstrated nematocidal activity against the nematode *Meloidogyne javanica* at a concentration of 100 ppm [79]. After 48 h of incubation, 100 mg/mL gold nanoparticles of *R. stricta* aqueous extract inhibited the growth of intra-THP-1 amastigotes (IC50: 1443 mg/mL) [50]. *R. stricta* extracts demonstrated the ability to suppress nutsedge density, length, and weight (fresh and dry) of the root and shoot [80]. *R. stricta* extract resulted in (91%) mortality in *Culex pipiens* by decreasing the expression level of acetylcholinesterase and glutathione S-transferase [81]. The ZnO nanoparticles and leaf extract of *Rhazya stricta* were revealed to be effective antimalarial agents at a 50% inhibitory concentration (IC50: 3.41 g/mL) [82]. When injected into the blood stream, an aqueous extract of the stems and roots was somewhat poisonous to American cockroaches but had no impact on German cockroaches or milkweed bugs [83].

## 12. Conclusions

For over 50 years, phytochemicals, pharmacological, and biological activities of *R. stricta* whole extract were the focus of attention in the Middle East and the South of Asia. *R. stricta* extract has been found to be toxic in animal models, as well as genotoxic and mutagenic in microorganism models, according to several studies. The phytochemistry profile of *R. stricta* contains a unique alkaloid content that has been isolated and identified significantly, and we found that the non-alkaloid contents need more investigation. *R. stricta* extract has shown pharmacological activity such as antimicrobial, anticancer, antidiabetic, and antioxidant activities, as well as biological activity such as insecticide, allelopathic, and soil remediation activities. Some pharmacological aspects, such as the antiviral activity of the plant extract have not been examined yet. Despite plenty of studies investigating *R. stricta* activity, only a few studies investigated the activity of its unique phytochemicals individually, so the advancement of research on the *R. stricta* plant should be moving from the whole extract level to the phytochemical levels.

## Figures and Tables

**Table 1:** *R. stricta* taxonomy according to Global Biodiversity Information Facility [7].

Kingdom	*Plantae*
Phylum	*Tracheophyta*
Class	*Magnoliopsida*
Order	*Gentianales*
Family	*Apocynaceae*
genes	*Rhazya Decne*
species	*Rhazya stricta Decne*

**Table 2:** List of *R. stricta* compounds identified and extracted using the PubChem data base [29].

Ref	CID	Compounds	
[2]	15558574	Akuammidine (Rhazine)	1.
[2]	5462421	Antirhine	2.
[2]	5491661	3-epi-Antirhine	3.
[2]	580281	Aspidospermidine	4.
[2]	5378963	Condylocarpine	5.
[2]	164952	Dihydrocorynantheol	6.
[2]	6857502	Eburnamenine	7.
[2]	101699	Eburnamine	8.
[2]	92112	Eburnamonine	9.
[2]	5280491	Geissoschizine	10.
[2]	6436828	Isositsirikine	11.
[2,30]	5377267	16-Epi-Z-isositsirikine	12.
[2]	125060	Leuconolam	13.
[2]	160263	Rhazinilam	14.
[2]	169527	Tetrahydrosecamine	15.
[2]	193109	Presecamine	16.
[2]	5458504	Sewarine	17.
[2]	10066724	Stemmadenine	18.
[2]	301805	Strictamine	19.
[2]	10345799	Strictosamide	20.
[2]	161336	Strictosidine	21.
[2]	20485	Tabersonine	22.
[2]	72340	Tetrahydroalstonine	23.
[2]	5384527	Vallesiachotamine	24.
[2]	6443646	Rhazimine	25.
[2]	14109838	Rhazimanine	26.
[2,30]	5318674	Bhimberine	27.
[2]	101607204	Leepacine	28.
[2]	12314912	Rhazidigenine	29.
[2,30]	624708	(+)-Quebranchamine	30.
[2,30]	579873	(-)-Vincadiformine	31.
[2]	102276348	Secamine	32.
[2]	12444819	Vincadine	33.
[2]	101407506	Strictamine-N-oxide	34.
[2]	624448	1,2-Didehydroaspidospermidine	35.
[2]	102276826	Dihydrosecamine	36.
[2]	102276822	Dihydropresecamine	37.
[2,30]	626805	Rhazidigenine-N-oxide	38.
[2,30]	14825828	Decarbomethoxy-15,20,16,17-tetrahydrosecodine	39.
[2]	624449	Dihydroeburnamenine	40.
[2,30]	5757451	Nor-C-luorocurarine	41.
[2]	5374154	Polyneuridine	42.
[2,30]	540749	(−)-16R,21R-Omethyleburmanine	43.
[2,30]	94255	(-)-Vincadifformine	44.
[2,30]	---------------	Aspidospermiose	45.
[2,30]	----------------	Bhimberine-N-oxide	46.
[2,30]	----------------	Rhazicine	47.
[2,30]	----------------	2-Methoxy 1-2, dihydrorhazamine	48.
[2,30]	----------------	16-Hydrorhazisidine	49.
[2,30]	----------------	Dihydrosecodine	50.
[2,30]	----------------	HR-1	51.
[2,30]	----------------	N-methylleuconolam	52.
[2,30]	----------------	Rhazinaline	53.
[2,30]	----------------	Stricticine	54.
[2,30]	----------------	Strictalamine	55.
[2,30]	----------------	Strictigine	56.
[2,30]	----------------	Strictisidine	57.
[2,30]	----------------	Tetrahydrosecodine	58.
[2,30]	----------------	Vincanicine	59.
[2,30]	----------------	Isorhazicine	60.
[2,30]	----------------	Rhazinol	61.
[2,30]	----------------	Rhazimol	62.
[2,30]	----------------	Rhazizine	63.
[2,30]	----------------	15-Hydroxyvincadifformine	64.
[2]	177185	16s,16′-Decarboxytetrahydrosecamine	65.
[2,30]	--------------	Strictibine	66.
[2]	101967159	Rhazimal	67.
[2]	12313538	Vincanine	68.
[2]	5280794	Stigmasterol	69.
[2]	222284	β -Sitosterol	70.
[2]	20756463	Phytochelatins	71.
[2]	--------------	Bis-strictidine	72.
[2]	--------------	1,2-Dehydroaspidospermine N-oxid	73.
[2]	--------------	3, 14-Dehydrorhazigine	74.
[2]	--------------	Dihydroebumamenine	75.
[2]	--------------	21S-Ebumamenine	76.
[2]	--------------	16-Formylstrictamine	77.
[2]	--------------	Harhingine	78.
[2]	11530478	15β-Hydroxyvincadifformine	79.
[2]	--------------	16,hydroxyrhazisidine	80.
[2]	6442678	Isovallesiachotamine	81.
[2]	--------------	2-Methoxy 1,2- dihydorhazimine	82.
[2]	--------------	17-Methoxy 1, 17-dihydorhazimine	83.
[2]	--------------	16R,21R-O-Methylebumamine	84.
[2]	--------------	N-Methyleuconulam	85.
[2]	5581319	Norfluorocurarine	86.
[2]	--------------	Nβ-methyl strictamine	87.
[2]	--------------	Rhazigine	88.
[2]	--------------	Rhazimidine	89.
[2]	--------------	Rhazinol	90.
[2]	--------------	Rhazind	91.
[2,30]	21725847	Rhazinine	92.
[2]	--------------	Rhazisidine	93.
[2]	--------------	Strictanine	94.
[2]	--------------	Strictibine	95.
[2]	--------------	Stricticine	96.
[2]	--------------	Strictimine	97.
[2]	--------------	Strictimidine	98.
[2]	--------------	Strictine	99.
[2]	--------------	Tetrahydropresecamine	100.
[4]	5280343	Quercetin	101.
[4]	72281	Hesperitin	102.
[4]	5280863	Kaempferol	103.
[4]	5280459	Quercetin-3-rhamnoside	104.
[4]	5280804	Isoquercetin	105.
[4]	5280805	Rutin	106.
[4]	5280443	Apigenin	107.
[4]	5280445	Luteolin	108.
[4]	5280637	Luteolin-7-glucoside	109.
[4]	5280442	Acacetin	110.
[4]	5280441	Apigenin-8-C-glucoside	111.
[27]	41961	Tetrahydro-2-(12-pentadecynyloxy)-2H-pyran	112.
[27]	14276	Azocine, octahydro-	113.
[27]	138546	8-Azabicyclo[3.2.1]oct-2-ene	114.
[27]	580053	2-Amino-6-methoxypyridine	115.
[27]	573816	4(1H)-Pyridinone, 2,3-dihydro-1-methyl-	116.
[27]	23494	Tetratetracontane	117.
[27]	19901	Pyridine, 3-ethyl-5-methyl-	118.
[27]	61038	1,3-Propanediol, 2-butyl-2-ethyl-	119.
[27]	86541	Nonane, 4,5-dimethyl-	120.
[27]	11006	Hexadecane	121.
[27]	545627	Dodecane, 4,6-dimethyl-	122.
[27]	20282	Dodecane, 1-iodo-	123.
[27]	520211	1-(3-Aminopropyl)-2-pipecoline	124.
[27]	545941	2-Isopropyl-5-methyl-1-heptanol	125.
[27]	93447	Dodecane, 2,7,10-trimethyl-	126.
[27]	285814	4,4′-Isopropylidenebis(3-methyl-2-isoxazolin-5-one)	127.
[27]	541883	Cyclohexanamine, N-methyl-n-propyl-	128.
[27]	587705	Acetamide, 2-(3-hydroxy-8-aza-bicyclo[3.2.1]oct-8-yl)-N-(2,4,6-trimethylphenyl)-	129.
[27]	73559	1H-Isoindole-1,3(2H)-dione, hexahydro-	130.
[27]	11636	Heptacosane	131.
[27]	545611	Decane, 2,3,5,8-tetramethyl-	132.
[27]	14536	Quinoline, 2,4-dimethyl-	133.
[27]	520709	Eicosane, 1-iodo-	134.
[27]	7311	2,4-Di-tert-butylphenol	135.
[27]	14845381	((8R,8aS)-8-Isopropyl-5-methyl-3,4,6,7,8,8a-hexahydronaphthalen-2-yl)methanol	136.
[27]	33865	11-Methyldodecanol	137.
[27]	95337	1-Decanol, 2-hexyl-	138.
[27]	549960	Cyclohexane, 1-ethyl-2-propyl-	139.
[27]	67043	1-Naphthalenamine, N-ethyl-	140.
[27]	95997	3′,5′-Dimethoxyacetophenone	141.
[27]	3518	Guanethidine	142.
[27]	41209	Heptadecane, 2,6,10,15-tetramethyl-	143.
[27]	598127	Pyridine, 5-methyl-4-phenyl-	144.
[27]	5281520	Humulene	145.
[27]	12798926	2-Bromotetradecane	146.
[27]	20831	2-Tetradecanol	147.
[27]	91693137	Carbonic acid, eicosyl vinyl ester	148.
[27]	543807	1,7-Dimethyl-4-(1-methylethyl)cyclodecane	149.
[27]	542202	Methoxyacetic acid, 2-pentadecyl ester	150.
[27]	9603606	Thiourea, (5,5-dimethyl-3-oxo-5,6-dihydropyrrolo[2,1-a]isoquinolin-2-ylidene)-	151.
[27]	581546	Sydnone, 3-(2-naphthyl)-	152.
[27]	152961	Ethanone, 2-chloro-1H-indol-1-yl-	153.
[27]	5357283	2-Propenoic acid, 3-(4-hydroxy-3-methoxyphenyl)-,methyl ester	154.
[27]	598515	1H-Indole, 4-(3-methyl-2-butenyl)-	155.
[27]	22833370	1,2-Benzenedicarboxylic acid, bis(2-ethylpropyl)ester	156.
[27]	2936295	Pyrrolidine-2,5-dione, 1-(3-chlorophenyl)-3-(4-phenyl-3,6-dihydro-2H-pyridin-1-yl)-	157.
[27]	42647321	(6-methylquinolin-2-yl)methanamine	158.
[27]	8181	Hexadecanoic acid, methyl ester	159.
[27]	598481	1,7-Dimethylene-2,3-dimethylindole	160.
[27]	62603	Benzenepropanoic acid, 3,5-bis(1,1-dimethylethyl)-4-hydroxy-, methyl ester	161.
[27]	58527531	Isobutyl tetradecyl ether	162.
[27]	5364506	trans-13-Octadecenoic acid, methyl ester	163.
[27]	5364509	9-Octadecenoic acid (Z)-, methyl ester	164.
[27]	8201	Methyl stearate	165.
[27]	5280450	9,12-Octadecadienoic acid (Z,Z)-	166.
[27]	614424	3-Amino-5-chloro-benzofuran-2-carboxylic acid methyl ester	167.
[27]	545593	Heptacosane, 1-chloro-	168.
[27]	5376940	O-(2,4-Dinitrostyryl)-phenol	169.
[27]	9548854	Aspidofractinine	170.
[27]	91693138	Carbonic acid, octadecyl vinyl ester	171.
[27]	292723	Heptadecane, 8-methyl-	172.
[27]	14259	Eicosanoic acid, methyl ester	173.
[27]	292285	Octadecane, 3-ethyl-5-(2-ethylbutyl)-	174.
[27]	76958	Oxiranedodecanoic acid, 3-octyl-, cis-	175.
[27]	91712839	Fumaric acid, monoamide, N,N-dimethyl-, 1-naphthyl ester	176.
[27]	624530	.beta.-Hydroxyquebrachamine	177.
[27]	7641	Hexanedioic acid, bis(2-ethylhexyl) ester	178.
[27]	594104	5,8-Dimethylquinoxaline	179.
[27]	580937	Acetic acid, 6-morpholin-4-yl-9-oxobicyclo[3.3.1]non-3-yl ester	180.
[27]	1969543	1,2,5-Oxadiazole-3-carboxamide, 4-amino-N-[2-[[(3-chlorophenyl) methyl] amino] ethyl]-	181.
[27]	580956	Naphtho[1,2-b]furane-2,8-dione, 2,3,3a,4,5,5a,8,9b-octahydro-9-methyl-3-(3,3-dimethyl-1-piperidylmethyl)-	182.
[27]	252320	(+/-)-Uleine	183.
[27]	5373573	Apparicine, Nb-methyltetrahydro-	184.
[27]	112885	Octadecanoic acid, 3-oxo-, ethyl ester	185.
[27]	593916	2,20-Cyclo-8,9-secoaspidospermidine, 3-methyl-, (2.alpha.,3.beta.,5.alpha.,12.beta.,19.alpha.,20R)-	186.
[27]	579942	1-Methyl-16-methoxyaspidospermidin-4-one	187.
[27]	58184953	12H-benzo[b]phenoxazine, 12-methyl-	188.
[27]	20619411	Methyl 8-methyl-nonanoate	189.
[27]	610181	2-Methyl-7-phenylindole	190.
[27]	619344	Eburnamenin-14-ol, 14,15-dihydro-, (14.beta.)-	191.
[27]	91712719	l-Alanine, n-propargyloxycarbonyl-, ethyl ester	192.
[27]	10949	2,4-Diamino-6-methyl-1,3,5-triazine	193.
[27]	620161	Indolo[2,3-a]uinolizine-4(12H)-one, 1,2,3,6,7,12b-hexahydro-3,12b-dimethyl-	194.
[27]	580315	Aspidospermidine, 1-ethyl-	195.
[27]	201188	Vincaminol	196.
[27]	425980	Cleavamine	197.
[27]	91719594	Phthalic acid, 2-ethylbutyl nonyl ester	198.
[27]	590836	Phthalic acid, bis(7-methyloctyl) ester	199.
[27]	638072	Squalene	200.
[27]	71204	Apovincamine	201.
[31]	15376	Vincamine	202.
[31]	64971	Betulinic acid	203.
[31]	382831	Pomolic acid	204.
[31]	73659	Maslinic acid	205.
[31]	21676297	Kaempferol rhamnoside rutinoside	206.
[31]	64945	Ursolic acid	207.
[31]	12313704	Oleanolic acid	208.
[31]	8969	Yohimbine	209.
[32]	998	Phenylacetaldehyde	210.
[32]	28111	(Dimethylamino)methylene malononitrile	211.
[32]	5283356	Trans-2-Undecenal	212.
[32]	12266719	Dihydrocitronellal	213.
[32]	62321	Linalyl butyrate	214.
[32]	439507	D-Allose	215.
[32]	822800	1-(3,4-Dimethoxyphenyl) ethanone	216.
[32]	543855	2,2-Tricosenoic acid	217.
[32]	2537	Camphor	218.
[32]	3520	Guanidine	219.
[33]	11005	Myristic acid	220.
[33]	44256490	Pentadecylic acid	221.
[33]	985	Palmitic acid	222.
[33]	44256491	Margaric acid	223.
[33]	5281	Stearic acid	224.
[33]	10467	Arachidic acid	225.
[33]	8215	Behenic acid	226.
[33]	17085	Tricosylic acid	227.
[33]	11197	Lignoceric acid	228.
[33]	445638	Palmitoleic acid	229.
[33]	445639	Oleic acid	230.
[33]	5282761	Vaccenic acid	231.
[33]	5282768	Gondoic acid	232.
[33]	5281116	Erucic acid	233.
[33]	5280450	Linolic acid	234.
[33]	5280934	Linolenic acid	235.
[34]	441975	Ajmalicine	236.
[34]	100004	Tubotaiwine	237.
[34]	10314057	Akuammicine	238.
[34]	101688177	Fluorocarpamine	239.
[34]	624111	Decarbomethoxytabersonine	240.
[34]	73391	Serpentine	241.
[35]	334274	Tetrahydrosecamine diol	242.
[36]	--------------	Epi-rhazyaminine	243.
[36]	--------------	20-epi-sitsirikine	244.
[37]	85779	2-Hexadecanol	245.
[37]	8973	3-O-Methyl-d-glucose	246.

**Table 3:** Number of *R. stricta* compounds in each group.

Groups	No.
Alkaloid	118
Fatty acid	20
Flavonoid	12
Terpenes	9
Sterol	2
Peptides	1
Others	85

## Data Availability

All data included in the main text.
